# Production of vaccination videos in India: learnings from a science-art partnership

**DOI:** 10.1186/s12889-023-15607-w

**Published:** 2023-04-21

**Authors:** Julia Burleson, Rohini Ganjoo, Sidharth Rath, Nilambar Rath, Ananya Bhaktaram, Amelia M. Jamison, Neil Alperstein, Paola Pascual-Ferra, Daniel J. Barnett, Satyanarayan Mohanty, Manoj Parida, Peter Orton, Eleanor Kluegel, Rajiv N. Rimal

**Affiliations:** 1 Department of Health, Behavior and Society, Bloomberg School of Public Health, Johns Hopkins University, Baltimore, MD 21205 USA; 2 Department of Biomedical Laboratory Sciences, George Washington University, Ashburn, 20147 USA; 3 Swasthya Plus Network, Chandrasekharpur, , Bhubaneswar, 751017 Odisha India; 4 OdishaLIVE, Samanta Vihar, Bhubaneswar, 751017 Odisha India; 5 Department of Communication, Loyola University of Maryland, Baltimore, MD 21210 USA; 6 Department of Environmental Health & Engineering, Bloomberg School of Public Health, Johns Hopkins University, Baltimore, MD 21205 USA; 7 D-Cor (Development Corner) Consulting, Satya Nagar, Bhubaneswar, 751007 Odisha India; 8 Wellflix Inc, Hillsborough, NC 27278 USA

**Keywords:** Vaccination, COVID-19, Entertainment-education videos, Vaccine hesitancy, Messaging, Filmmaking collaboration

## Abstract

**Background:**

Cross-cultural communication, often conceptualized along culture and language dimensions, is an important issue for collaborative teams that include both scientists and artists. Such teams must balance the standardization needs of rigorous scientific methods, on the one hand, with openness for artistic creativity, on the other. The scientific literature does not provide clear guidance on how to structure such collaborations.

**Discussion:**

We created eight videos manipulating the type of appeal, message tone, and gender of the vaccine promoter in a 2 × 2 × 2 between-subjects experiment. The four stages of the filmmaking process were 1) conceptualizing filmmaking and script writing through a scientific lens, 2) pilot testing and finalizing the script, 3) video production and editing, and 4) dissemination. We describe the process and learnings from a collaboration that included filmmakers, researchers, and vaccine experts from India and the United States in producing, disseminating, and evaluating videos that promoted vaccine uptake in Odisha, India.

**Conclusions:**

When projects require close collaborations between scientists and artists, committing to a unified vision is essential for facilitating open, bidirectional communication and building trust between the partners. Clearly denoting research boundaries ensures that the scientific needs of the project are met while simultaneously welcoming space for the filmmakers’ creativity, fostering a sense of ownership, and enhancing the final product.

**Supplementary Information:**

The online version contains supplementary material available at 10.1186/s12889-023-15607-w.

## Background

More than six million people worldwide have died from COVID-19, underscoring the importance of effective public health communication for vaccinations [1]. In India, the government promotes vaccinations through television, radio, and social media [2]. Typically, state and local governments work with local filmmakers in a prescriptive manner, providing them with a script, budget, and predetermined vision. The government even publishes its procurement guidelines specifying payment amounts for health promotion videos of varying lengths [3].

Regulations can limit artistic expression, which may attenuate the effectiveness of health communication campaigns. In addition, the needs of rigorous scientific evaluations may add another restrictive layer to the overall process. Here we describe how we worked through these underlying issues in our project. We aimed to work with local filmmakers in Odisha, India to produce, disseminate, and evaluate videos promoting vaccination against COVID-19 [4]. Our research team, possessing scientific expertise in behavior change and health communication, collaborated with our creative team, possessing expertise in filmmaking and social media dissemination. With limited knowledge but extensive curiosity about the other party’s areas of expertise, we sought to balance free-flowing creativity with scientific rigor for evaluation. This paper is the result of a collaboration between a research team with scientific expertise in behavior change and health communication, on one hand, and filmmakers with narrative expertise in scriptwriting, on the other hand, who created videos promoting the second dose of the COVID-19 vaccines.

### Local context

India has reported over 43 million COVID-19 cases since the beginning of the pandemic [1]. Approximately 73% of the population have received their first dose, and 62% have received two doses of the vaccine [5]. Additionally, at the beginning of this study, an 11% gap existed between the proportion of people who had received two doses versus those who had received only the first dose [5]. Though the Indian government recommended a 16-week wait between the first and second doses, the large gap in vaccination is difficult to explain, as both doses are provided free of charge [6].

## Research study

As the world’s largest film producer, India’s entertainment industry has yet to be sufficiently tapped for promoting vaccinations. While most vaccination messages broadcast by the Indian government are structured as public service announcements, we utilized an approach to creating videos that would be different from the din of government-produced announcements. Here, we describe the lessons we learned from engaging in a cross-cultural collaboration between United States-based university researchers and India-based filmmaking experts to create and disseminate videos promoting COVID-19 vaccines.

### Filmmaking methodology

Our experiment investigated whether videos could change people’s attitudes, beliefs, and intentions to receive COVID-19 vaccines. We manipulated three variables: type of appeal (collectivistic or individualistic), message tone (humorous or serious), and the gender of the vaccine promoter (male or female). The resulting experiment, which adopted a 2 × 2 × 2 between-subjects design, produced eight different videos with similar scripts, camera angles, and post-production features, except for the experimental manipulations. As a sample, we have included the script pertaining to the male, serious, collective experimental manipulation (see Additional file 1) [4]. An additional document lists the YouTube links for all eight videos (see Additional file 2). Written consent was obtained from all participants involved in the study. The filmmaking process comprised 1) conceptualizing filmmaking and script writing through a scientific lens, 2) pilot testing and finalizing the script, 3) video production and editing, and 4) dissemination.

### Conceptualizing filmmaking and script writing through a scientific lens

We assembled a professional production team in Bhubaneshwar, India, comprising a national award-winning filmmaker, assistant director, director of photography, cast, and crew [4].

We adopted a narrative approach to create videos distinct from government-produced announcements [7]. Borrowing ideas from entertainment education, we adopted a narrative approach for the videos targeted to an 18 – 35-year-old population. The filmmakers depicted a common social event (a child’s birthday celebration) where vaccination issues arose incidentally and not as the primary thrust of the communication [8]. Embedded within the depiction of the birthday event were the various independent variable manipulations: discussions about the risks of COVID-19 and the benefits of vaccination were framed in either individual or collectivistic language, the overall tone was either funny or serious, and either a male or a female protagonist advocated for vaccinations.

The filmmaking team first developed a draft script in Odia, which was subsequently translated into English. The research group consulted with experts on the filmmaking team about the video plot and provided feedback from both scientific and narrative perspectives. To ensure minimal variance in video lengths across the videos, we scripted the research study manipulations into the dialogue to keep the total time for each dialogue block and the total number and lengths of different dialogue blocks similar [4].

### Pilot-testing and finalizing the script

We convened a community advisory board (CAB) comprising two young parental couples, a doctor, a school principal, and an accredited social health activist. The CAB reviewed four audio-recorded scripts: two humorous and two neutral, each featuring one individualistic and one collectivistic viewpoint [4]. The CAB feedback was generally positive with minor suggested edits. One significant feedback from the CAB, particularly for the humorous manipulation, centered around the need to strike a meaningful balance between the seriousness of the pandemic, which has resulted in significant numbers of deaths, and the study’s need to inject humor as a message feature. Further, the CAB was able to distinguish between the individualistic and collectivistic appeals, which indicated that the manipulation would likely be successful. We incorporated the CAB's feedback into the scripts before filming.

### Video production and editing

Formal features of videos– specific production techniques such as cuts, fades, zooms, and angles– are independent of the video message but impact the viewing experience [9–12]. We sought to keep formal features constant across the videos by dividing the videos into shots and filming similar shots for all videos together. Thus, we did not shoot each video from beginning to end before shooting the next video. Instead, we filmed all eight scenes at one camera angle before moving on to the next scene.

During editing, we selected shots for the first video and arranged them in the correct sequence (described in Table 1). Using the first video as a template, we only changed specific shots that were different due to the research manipulation for the remaining videos. This method ensured consistency across the videos. The filmmaking team, well-versed in the research needs and aims, subsequently reviewed the videos to ensure uniformity across non-manipulated content.

**Table 1:** Sample shot list for the first 30 seconds of four videos

TIME	REMARKS	VIDEOS			
		Female, Serious, Collective	Female, Comic, Collective	Male, Serious, Individual	Male, Comic, Individual
0:00–0:03	Same shot	Happy birthday card	Happy birthday card	Happy birthday card	Happy birthday card
0:04–0:05	Same shot	Cake is served	Cake is served	Cake is served	Cake is served
0:05–0:06	Same shot	Two men watch	Two men watch	Two men watch	Two men watch
0:07–0:08	Same shot	Cake is cut and served	Cake is cut and served	Cake is cut and served	Cake is cut and served
0:08–0:09	Same shot	Three women sit and watch	Three women sit and watch	Three women sit and watch	Three women sit and watch
0:10–0:12	Similar shot	Mini (female protagonist) walks toward the party	Mini (female protagonist) walks toward the party	Muna (male protagonist) walks toward the party	Muna (male protagonist) walks toward the party
0:12–0:14	Similar shot	Ambika (aunt) talks	Ambika (aunt) talks	Ambika (aunt) talks	Ambika (aunt) talks
0:14–0:15	Different shots (extra shots for comic)		Mini puts her bag down		Muna puts his bag down
0:16–0:16	Similar shot	Somnath (vaccine skeptic) looks at Mini	Somnath (vaccine skeptic) looks at Mini	Somnath (vaccine skeptic) looks at Muna	Somnath (vaccine skeptic) looks at Muna
0:17–0:18	Similar shot	Mini unzips the bag	Mini unzips the bag	Muna unzips the bag	Muna unzips the bag
0:18–0:19	Similar shot	Ambika walks with the cake in hand	Ambika walks with the cake in hand	Ambika walks with the cake in hand	Ambika walks with the cake in hand
0:19–0:20	Similar shot	Camera on Mini	Camera on Mini	Camera on Muna	Camera on Muna
0:21–0:23	Similar shot	Camera on Ambika as she serves cake	Camera on Ambika as she serves cake	Camera on Ambika as she serves cake	Camera on Ambika as she serves cake
0:23–0:24	Similar shot	Mini gives the gift and takes the cake	Mini gives the gift and takes the cake	Muna gives the gift and takes the cake	Muna gives the gift and takes the cake
0:25–0:26	Similar shot	Ambika smiles	Ambika smiles	Ambika smiles	Ambika smiles
0:26–0:28	Similar shot	Mini talks	Mini talks	Muna talks	Muna talks
0:28–0:29	Similar shot	Ambika talks	Ambika talks	Ambika talks	Ambika talks

### Dissemination

We disseminated the videos through the Swasthya Plus Network Odia website, Facebook page (513,000 subscribers), YouTube channel (248,000 subscribers), and WhatsApp groups in February 2022 [4].

## Filmmaking team response

This research project’s approach to filming public health campaigns was unlike any previous commercial assignment the filmmaking team had undertaken. Ensuring similarity between the eight videos was not initially intuitive to the filmmakers, as they often do not keep video elements consistent in their creative products. In the early phases of the project, the creative process– of freely navigating everyday realities to find stories that connect and communicate– was seemingly at odds with the deliberate, structured, and prescriptive research process.

However, after several virtual and in-person discussions with the research team, the filmmakers understood the scientific theories undergirding the researchers’ approach and incorporated this into their filmmaking process. The filmmaking team began viewing the research process not as constraints on creativity, but as sandboxes in which to fill stories. Instead of being at odds, science helped the filmmakers find relatable stories that communicated effectively with specific audiences and understand what made the stories impactful. Through the collaborative experience, the filmmakers tested the effectiveness of their creative products and experimented with centering the video’s target audience in the design process.

Ontological and epistemological differences can create challenges when bridging the worlds of the arts and sciences. The scientific process itself is based in a positivist epistemology, while the arts are classically associated with interpretivist and constructivist perspectives. As with all applied work, some nuances emerge in practice, but this central tension between art and science remains constant. Instead of viewing this as an obstacle, we focused on the creative potential of such tension, allowing for competing views to generate new avenues of thought. We recognized that there are no singular meanings inherent in a video, acknowledging that perception is shaped by the complex interactions between viewer and video. However, from a scientific perspective, the success of the experiment required each video to distinctly convey a specific emotional experience.

This was clearest with the element of humor. Humor itself is notoriously subjective, and perhaps there is nothing *less* funny than overexplaining the joke. Between these two poles, we were able to reach a place where we achieved nuanced humor through the creative use of local vernacular and wordplay; this has been published as a manipulation-check variable in a compendium study (Bhaktaram, 2022; masked for blind review). While such subtlety was initially a concern for the social science team, through our rigorous process of pre-testing and experimental design, we were able to demonstrate that viewers distinguished between the funny and serious videos, without having to push the humor into farce. Indeed, feedback from the community praised our ability to present humorous content on such a delicate topic as COVID-19, despite many in the community having experienced great personal hardship and loss. This success was only possible through partnership.

## Lessons learned

The intersection between the exploratory process of research and the storytelling aspect of filmmaking is complex. To create media that successfully answer a scientific question, researchers must facilitate open and clear communication to encourage a mutually beneficial and collaborative workspace. Researchers should discuss the objectives of the study, scientific theories informing the work, and justifications for study variables with the filmmakers in the partnership. These are not features of everyday parlance among filmmakers, including those with whom we collaborated for this project, but they are critical aspects of many research inquiries.

Researchers are also often not well-versed in filmmaking terminology or postproduction processes. Thus, when projects require close collaborations between the two parties, frequent and clear communication that solicits input from all stakeholders is important. Committing to a unified vision is essential for facilitating open and honest bidirectional communication and building trust between researchers and local partners. Further, to ensure seamless communication between researchers and filmmakers, particularly in transnational partnerships, written documentation of the evolving rationale and feedback surrounding the experiment are helpful to clarify, communicate, and coordinate. With the continuously changing landscape of COVID-19 variants, written records provided the research team with the evolving local context and the filmmaking team with clarity. Additionally, clearly denoting research boundaries ensures that the scientific needs of the project will be met while simultaneously inviting space for the filmmakers’ creativity, fostering a sense of ownership, and enhancing the final product.

## Conclusion

This project’s primary objective was to produce and disseminate videos through social media platforms. The importance of the experimental methodology was not immediately intuitive to the filmmakers. However, they supported the methodology after the researchers framed the benefit of variable isolation not only in terms of scientific rigor but also as a way for filmmakers to test the effectiveness of different video components. Expressing the benefits of the project approach for both the research and filmmaking teams are key to building strong and mutually beneficial relationships. When projects require close collaborations between researchers and local partners, committing to a unified vision and maintaining communication channels are essential for facilitating open and honest bilateral communication and building trust between researchers and local partners. Clearly expressing research objectives for the project ensures that its scientific needs will be met while giving the filmmakers leeway for creativity that creates a sense of ownership and strengthens the final product.

By collaboratively designing and creating health messaging content, cross-cultural research and filmmaking partnerships can lead to widely shared media products that resonate with the target population. The researchers contribute their scientific expertise in behavior change and health communication, while filmmakers contribute their narrative expertise in scriptwriting. Engaging local filmmakers in both the planning and implementation stages helps foster ownership of the content and creates appealing media for society that aims to nudge them toward healthy behaviors.

## Supplementary Information

**Additional file 1.** Male, serious, collective video script.

**Additional file 2.** Production of vaccination videos in India: learnings from a science-art partnership video links.

## Data Availability

Data sharing is not applicable to this article as no datasets were generated or analyzed during the current study.

## Abbreviation

CAB: Community Advisory Board
