# Supplementary material for: Production of vaccination videos in India: learnings from a science-art partnership
Source: BMC Public Health. 2023 Apr 21;23:736. doi: 10.1186/s12889-023-15607-w (PMC10119828; doi:10.1186/s12889-023-15607-w)
Supplement: Supplementary file 1 — Additional file 1. Male, serious, collective video script. [file 12889_2023_15607_MOESM1_ESM.docx]

**FADE IN:**

EXT. VILLAGE HOME - INNER COURTYARD - DAY

Light MUSIC. The courtyard and its central tree are decorated with paper flags, flowers, and balloons. PEOPLE, in their 20s and 40s, happily socialize. At a table AMBIKA, 40, is cutting a cake. Slices sit on paper plates.

EXT. THROUGH COURTYARD ENTRANCEWAY

MUNA, 25, goes and stops at the entrance, looks in, then enters. Ambika looks up, sees Muna, and smiles.

AMBIKA

Oh Muna, you’re late!

REVEAL banner hanging beyond Ambika: SURAJ 9^th^ Birthday Celebration. Muna removes a wrapped gift from his backpack.

MUNA

But, where is my nephew, Suraj?

AMBIKA

Oh, He’s off playing with

Friends after cutting the cake. Here, have some cake.

Muna hands the gift to Ambika, and takes the plate of cake.

MUNA

I have to go, sister.

AMBIKA

Where are you headed in such a hurry?

MUNA

My parents and I are all getting our second COVID vaccine today at the hospital.

AMBIKA

Can’t you go tomorrow?

INTERCUT -- OTHER PARTYGOERS

overhearing this conversation..

BACK TO SCENE

MUNA

No, our appointment is for today.

SOMANATH, 35, who’s been listening, steps forward.

SOMANATH
(sarcastically)

So, Muna, your family has already got the first shot – why more! And your family’s body immunity must be enough!

MUNA

No. If that were true, Somanath brother, why are so many unvaccinated people dying from Covid?

And – haven’t you got all the shots?

SOMANATH

Just one. Under pressure from my wife, Anjali.

WIDER TO INCLUDE ANJALI (HIS WIFE)

who stands alongside Somanath. She grimaces in irritation.

SOMANATH(Continuing)

No more shots for me!

AMBIKA

Then you prefer sickness and death, over getting the shot?

INTERCUT - OTHERS

Overhearing, reacting, now listening carefully.

BACK TO SCENE

MUNA

My doctor said when we’ve had all shots, we’ll be less likely to get COVID or pass it to others. I can be less worried about passing COVID to my family when I come home from work!

AMBIKA

You’re right, Muna! Go, Get your family and your 2^nd^ shots!

MUNA

I’ll get going, sister.

WIDER ANGLE - INCLUDE ANJALI

ALL Others are turning to look at Somanath.

MUNA (to Somanath, gently)

Brother, Please try to understand.

CLOSE - SOMANATH

Realizing others’ looks and knows he’s wrong. Finally…

SOMANATH

(weakly, dismissively)

What!?..

FADE OUT
