# Supplementary material for: Production of vaccination videos in India: learnings from a science-art partnership
Source: BMC Public Health. 2023 Apr 21;23:736. doi: 10.1186/s12889-023-15607-w (PMC10119828; doi:10.1186/s12889-023-15607-w)
Supplement: Supplementary file 2 — Additional file 2. Production of vaccination videos in India: learnings from a science-art partnership video links. [file 12889_2023_15607_MOESM2_ESM.docx]

**Production of Vaccination Videos in India: Learnings from a Science-Art Partnership Video Links**

| **Video** | **YouTube Link** |
| --- | --- |
| Video 1- Male, Serious, Collective | [https://youtu.be/V8lYT14XiiA](https://nam02.safelinks.protection.outlook.com/?url=https%3A%2F%2Fyoutu.be%2FV8lYT14XiiA&data=05%7C01%7Cjburles4%40jhu.edu%7C8a40f2fa7e4c4c1e3a4408da4ed5e952%7C9fa4f438b1e6473b803f86f8aedf0dec%7C0%7C0%7C637908977903431978%7CUnknown%7CTWFpbGZsb3d8eyJWIjoiMC4wLjAwMDAiLCJQIjoiV2luMzIiLCJBTiI6Ik1haWwiLCJXVCI6Mn0%3D%7C3000%7C%7C%7C&sdata=%2BA2SQC%2FLeoJ2Lwrw1DnBkRmNAo7oJ%2Bf0G%2FURwXbr%2Fx8%3D&reserved=0) |
| Video 2- Male, Serious, Individual | [https://youtu.be/Ldy2ZuVsyKs](https://nam02.safelinks.protection.outlook.com/?url=https%3A%2F%2Fyoutu.be%2FLdy2ZuVsyKs&data=05%7C01%7Cjburles4%40jhu.edu%7C8a40f2fa7e4c4c1e3a4408da4ed5e952%7C9fa4f438b1e6473b803f86f8aedf0dec%7C0%7C0%7C637908977903431978%7CUnknown%7CTWFpbGZsb3d8eyJWIjoiMC4wLjAwMDAiLCJQIjoiV2luMzIiLCJBTiI6Ik1haWwiLCJXVCI6Mn0%3D%7C3000%7C%7C%7C&sdata=aZfWyf424Ivf0haQZJ1mdTwLUvMKjU6GPcEFcZk13sw%3D&reserved=0) |
| Video 3- Male, Comic, Collective | [https://youtu.be/2zFZM9NjioU](https://nam02.safelinks.protection.outlook.com/?url=https%3A%2F%2Fyoutu.be%2F2zFZM9NjioU&data=05%7C01%7Cjburles4%40jhu.edu%7C8a40f2fa7e4c4c1e3a4408da4ed5e952%7C9fa4f438b1e6473b803f86f8aedf0dec%7C0%7C0%7C637908977903431978%7CUnknown%7CTWFpbGZsb3d8eyJWIjoiMC4wLjAwMDAiLCJQIjoiV2luMzIiLCJBTiI6Ik1haWwiLCJXVCI6Mn0%3D%7C3000%7C%7C%7C&sdata=zXfg8TObKdlaWeVlq4gzfkbtGz744OCheMP75aEsl0I%3D&reserved=0) |
| Video 4- Male, Comic, Individual | [https://youtu.be/UO8350oa6l0](https://nam02.safelinks.protection.outlook.com/?url=https%3A%2F%2Fyoutu.be%2FUO8350oa6l0&data=05%7C01%7Cjburles4%40jhu.edu%7C8a40f2fa7e4c4c1e3a4408da4ed5e952%7C9fa4f438b1e6473b803f86f8aedf0dec%7C0%7C0%7C637908977903431978%7CUnknown%7CTWFpbGZsb3d8eyJWIjoiMC4wLjAwMDAiLCJQIjoiV2luMzIiLCJBTiI6Ik1haWwiLCJXVCI6Mn0%3D%7C3000%7C%7C%7C&sdata=qgaWe68U53rn4Zzb7RyqWgt16OR3LmkXWWCzC7UwIoM%3D&reserved=0) |
| Video 5- Female, Serious, Collective | <https://youtu.be/hqMPFO0pEP0> |
| Video 6- Female, Serious, Individual | [https://youtu.be/9xyEL4otrhE](https://nam02.safelinks.protection.outlook.com/?url=https%3A%2F%2Fyoutu.be%2F9xyEL4otrhE&data=05%7C01%7Cjburles4%40jhu.edu%7C8a40f2fa7e4c4c1e3a4408da4ed5e952%7C9fa4f438b1e6473b803f86f8aedf0dec%7C0%7C0%7C637908977903275738%7CUnknown%7CTWFpbGZsb3d8eyJWIjoiMC4wLjAwMDAiLCJQIjoiV2luMzIiLCJBTiI6Ik1haWwiLCJXVCI6Mn0%3D%7C3000%7C%7C%7C&sdata=QXLtpaaQVTq18EzPkqABSvy1PytzCr4eLS440x02Yjw%3D&reserved=0) |
| Video 7- Female, Comic, Collective | [https://youtu.be/AHiikvtkHVk](https://nam02.safelinks.protection.outlook.com/?url=https%3A%2F%2Fyoutu.be%2FAHiikvtkHVk&data=05%7C01%7Cjburles4%40jhu.edu%7C8a40f2fa7e4c4c1e3a4408da4ed5e952%7C9fa4f438b1e6473b803f86f8aedf0dec%7C0%7C0%7C637908977903431978%7CUnknown%7CTWFpbGZsb3d8eyJWIjoiMC4wLjAwMDAiLCJQIjoiV2luMzIiLCJBTiI6Ik1haWwiLCJXVCI6Mn0%3D%7C3000%7C%7C%7C&sdata=WuchdpAZlHr6t3j4mRjl6m1poLCHOPmASBV5tcEcj2w%3D&reserved=0) |
| Video 8- Female, Comic, Individual | [https://youtu.be/YOOSWHH4gmU](https://nam02.safelinks.protection.outlook.com/?url=https%3A%2F%2Fyoutu.be%2FYOOSWHH4gmU&data=05%7C01%7Cjburles4%40jhu.edu%7C8a40f2fa7e4c4c1e3a4408da4ed5e952%7C9fa4f438b1e6473b803f86f8aedf0dec%7C0%7C0%7C637908977903431978%7CUnknown%7CTWFpbGZsb3d8eyJWIjoiMC4wLjAwMDAiLCJQIjoiV2luMzIiLCJBTiI6Ik1haWwiLCJXVCI6Mn0%3D%7C3000%7C%7C%7C&sdata=XCnX9ezLK%2BYDXGz3bCpGHwHr26xgh77UIEQOAxIq9H4%3D&reserved=0) |
